# Microevolutionary change in viscerocranial bones under congeneric sympatry in the Lake Tanganyikan cichlid genus *Tropheus*

**DOI:** 10.1007/s10750-021-04536-7

**Published:** 2021-02-22

**Authors:** Michaela Kerschbaumer, Lisbeth Postl, Christian Sturmbauer

**Affiliations:** 1 Institute of Biology, University of Graz, Universitätsplatz 2, 8010 Graz, Austria; 2 Library Feldkirch, Widnau 2-4, 6800 Feldkirch, Austria

**Keywords:** Geometric morphometrics, Semilandmarks, Sexual dimorphism, Mouthbrooders, Adaptation, Geomorph

## Abstract

The endemic Lake Tanganyika cichlid genus *Tropheus* lives at rocky shores all around the lake and comprises six species which are subdivided into about 120 morphologically similar but color-wise distinct populations. Typically, they live without a second *Tropheus* species, but there are some regions where two or even three sister species live in sympatry. We previously showed that there are morphological differences concerning head shape, eye size and insertion of fins among populations living alone compared to those living in sympatry with a second *Tropheus*. This study goes one step further to test if sympatry affects the shape of viscerocranial bones. By means of geometric morphometrics, we compare the shape of four bones among thirteen *Tropheus* populations, some of which in sympatry and some living alone. We quantify patterns of shape variation and estimate morphological disparity among the four bony elements in the study species and populations. We found consistent differences in the shape of one bony element among non-sympatric and sympatric populations, besides an extensive variation in the shape of viscerocranial bones within and among species. Furthermore, sexual dimorphism in *Tropheus* is clearly evident in the viscerocranial bones analyzed. We suggest that the relatively subtle morphological signal in sympatric vs. non-sympatric *Tropheus* populations is owed to the fact that the depth segregation does not yet represent a full shift in the trophic niche, albeit our data confirm that differences in ecologically relevant traits, such as bones of the preorbital region, play an important role in the process of niche separation and in the context of explosive diversification of cichlid fishes.

**Supplementary Information:**

The online version contains supplementary material available at 10.1007/s10750-021-04536-7.

## Introduction

Evolutionary biologists focus on factors promoting adaptation and their role in speciation. These can be most efficiently studied in species-rich communities, in which coexistence is facilitated by differential eco-morphological specialization (Losos, 2000; Gillespie, 2004; Losos et al., 2006; Reding et al., 2009). Morphological traits and their evolution play an important role in species diversification. It is not astonishing that adaptive radiation has been studied extensively in a wide variety of biological groups, such as reptiles (Losos et al., 1998, Butler et al., 2007), birds (Grant, 1981; Grant & Grant, 2002; Lovette et al., 2002; Petren et al., 2005; Reding et al., 2009; Lerner et al., 2011), land snails (Cowie, 1992; Goodacre & Wade, 2001; Parent & Crespi, 2009; Hoso, 2012) and fish (Kocher, 2004; Seehausen, 2004, 2006; Turner, 2007; Muschick et al., 2012). Phenotypic variation among individuals is triggered by the combination of phenotypic plasticity and genetically based differences. Phenotypic plasticity may play a role in colonization of new environments if plasticity causes changes in phenotypes that allow the population to survive under novel conditions, as suggested by the flexible stem hypothesis (West-Eberhard, 2003; Levis & Pfennig, 2016).

The review of Seehausen & Wagner (2014) concerning speciation, based on transitions between marine and freshwater habitats, transitions between discrete freshwater habitats, and ecological transitions within habitats, as well as speciation without distinct niche shifts gives a timely overview of the scope of studies on fish speciation. The assemblages of cichlid fish in East African Great Lakes, Victoria, Malawi, and Tanganyika are perfect model systems for many of those scenarios (Fryer & Iles, 1972; Kocher, 2004). The evolution of several distinct trophic specializations is apparently a key element to the ecological success of lacustrine cichlids (Greenwood, 1984) and the entire suspensorium is modified markedly among different foraging types, while changes in overall body shape remain subtle (Chakrabarty, 2005). Aside of the shape and type of oral and pharyngeal dentition, which can evolve in parallel in different radiations and even within a lake (Muschick et al., 2012), the shape of the preorbital region emerged as a key factor facilitating the impressive radiation of Lake Tanganyika (LT) cichlids (Cooper et al., 2010; Wanek & Sturmbauer, 2015). It was Cooper et al. (2010) who proposed that characterizing those changes in cichlid trophic morphology, which have contributed to adaptive radiation, allows for general evolutionary implications relevant to several other systems.

Already, there are a couple of studies dealing with this topic. For example, differences in certain bony elements of the viscerocranium were attributed to alternative trophic specialization (Albertson et al., 2005) and divergent selection was supposed to be involved in pushing rapid morphological divergence in the oral and pharyngeal jaws, which were suggested to be two modules that varied relatively independently among the emerging species (Liem, 1978; Powder & Albertson, 2015). Albertson et al. (2003b) established that the oral jaw apparatus is controlled by relatively few genes and some parts such as tooth shape by only a single gene, so that an extremely rapid response to selection is possible. Meanwhile, the involvement of a series of key developmental genes is implicated in this process (Roberts et al., 2011; Hu & Albertson, 2014; Parsons et al., 2014; Powder et al., 2014; Ahi, 2016) and a more integrative understanding of explosive diversification events is emerging through the rapidly progressing field of cichlid fish genomics, as reviewed by Salzburger (2018).

The species of the endemic Lake Tanganyika cichlid fish genus *Tropheus* Boulenger (1898) are highly specialized littoral algae scrapers at rocky shores. Currently, six species are described, one of which (*Tropheus duboisi* Marlier, 1959) represents a deep and eco-morphologically distinct offshoot, and the remaining five being closely related entities (Poll, 1986; Konings & Dieckhoff, 1992; Schupke, 2003). These are subdivided further into about 120 colorationally distinct but morphologically similar populations, most of which live in allopatry but some in sympatry (Konings, 2013). Although we know that a thorough revision of the genus is in preparation, we refer to formally described species throughout this study. *Tropheus* turned out to be an ideal model to study population differentiation and speciation (Sturmbauer & Meyer, 1992; Egger et al., 2007). First morphology has been considered to be highly similar among allopatric populations and sister species within the genus, most probably due to the fact that *Tropheus* is part of a complex species community in which it occupies the same niche in all allopatric habitats. Unexpectedly, several studies showed that there are differences between populations in body shape and even in single viscerocranial elements. All those differences mostly occur in ecologically relevant traits, so that microevolutionary change with an adaptive background seems likely (Maderbacher et al., 2008; Postl et al., 2008; Kerschbaumer et al., 2011). The first investigation concerning sexual dimorphism within *Tropheus* was carried out by Herler et al. (2010) where sexual dimorphism was investigated through geometric morphometrics. Shape differences among sexes were assessed in relation to the differentiation of populations and species. Shape variation between populations and sexes was primarily located in the cranial region. Sex-specific shape differences comprised a larger buccal area in females and can be explained as adaptation to maternal mouthbrooding. Population-specific differences mainly involve the position of the mouth, as a result of different ecological selection regimes in different habitats.

Within Lake Tanganyika, *Tropheus* populations occupy rocky shores with a wide range of water depths, sometimes down to 40 m. The highest individual density is found between 0.5 and 5 m depth (Kohda & Yanagisawa, 1992; Sturmbauer et al., 2008) possibly due to the optimum in algae productivity at this depth range. Kerschbaumer et al. (2014) studied the effects of partial co-occurrence of the ‘Ikola’ and ‘Kirschfleck’ populations of *Tropheus moorii* Boulenger, 1898 with *Tropheus polli* Axelrod, (1977). When sympatric *T. polli* primarily occupies the uppermost section of the rocky habitat, *T. moorii* lives at the deeper sections of the rocky littoral zone between about 3 m and 5 m. When *T. moorii* lives alone, it occupies the entire depth range. The study found significant morphological separation between non-sympatrically and sympatrically living *Tropheus* populations and linked them to an adaptation to environmental features at greater water depth and light transmission. Sympatric populations had a relatively smaller head, smaller eyes and a more anterior insertion of the pectoral fin. Concerning total shape variance, they revealed a significantly smaller within-population variance in *T. polli* populations than that of all *T. moorii* populations and a smaller variance in sympatric populations than in the non-sympatric populations. They came to the conclusion that could be result of stabilizing selection pressure owning to food competition of *T. moorii* with *T. polli* and other fishes in deeper water. Genetically, non-sympatric and sympatric ‘Ikola’ populations clustered together. *Tropheus* ‘Kirschfleck’ and *T. polli* were clearly distinct. They suggested that natural selection acts on both phenotypic plasticity and heritable traits and that both factors contribute to observed shape differences.

In our present work, we take a closer look on differences concerning the head and now focus on single bony elements. This study should extend the present knowledge by looking at a smaller (micro-) evolutionary scale. Among 13 different *Tropheus* populations, in sympatric and non-sympatric situations, we compared the shape of four viscerocranial bones, namely articular, lacrymale, preopercle and quadrate. These bony elements are nearly plane, so that two-dimensional landmarks are suitable for shape analysis. We investigated if morphological differences among species, color morphs, populations and sex could be found in the shape of single bony elements. By means of geometric morphometrics, we tested the hypothesis that shape differences concerning the head region between non-sympatrically and sympatrically living *Tropheus* populations are also reflected in the shape of single viscerocranial bones.

## Materials and methods

### Study populations

**Fig. 1:**
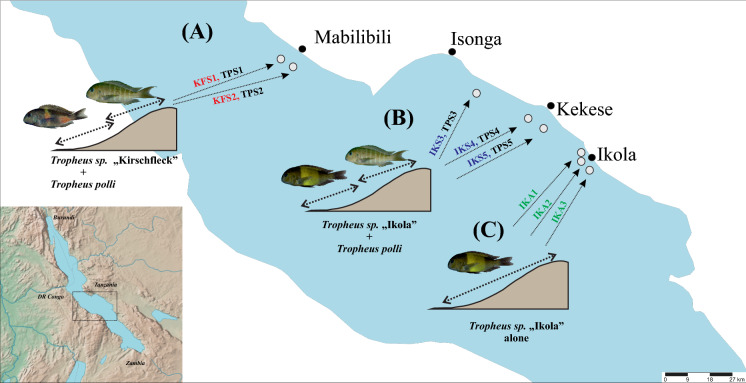
Sampling localities at Lake Tanganyika **A** *Tropheus*“Kirschfleck” in sympatry with *T. polli* near Mahale (KFS1) and Mabilibili (KFS2); **B** *Tropheus* “Ikola” in sympatry with *T. polli* from south of Isonga (IKS3), north of Kekese (IKS4), and at Kekese (IKS5); sympatric *T. polli* (TPS1-TPS5); **C** non-sympatric *Tropheus*“Ikola” living alone without a second *Tropheus* north of Ikola (IKA1), at Ikola (IKA2), and south of Ikola (IKA3). Living in sympatry can be equated with living in deeper habitat for *Tropheus moorii*, albeit with some overlap

Specimens of two species of the genus *Tropheus* (*T. moorii* and *T. polli*) were sampled from eight locations on the eastern coast of Lake Tanganyika (Fig. 1). At three locations, one species, i.e., *T. moorii* of the geographical variant ‘*Tropheus* Ikola’, occupied the full range of the preferred habitat (termed ‘non-sympatric *Tropheus* Ikola’), and at five locations, populations shared their habitat with a sister species, *T. polli* (termed ‘sympatric *Tropheus* Ikola’ and ‘sympatric *Tropheus* Kirschfleck’). We refer to these two situations as “habitat scenarios” throughout the study and use standardized abbreviations for populations as given in Fig. 1 and Table 1. Please note that the geographical variant (color morph) ‘*Tropheus* Ikola’ is synonymous with ‘*Tropheus* Kaiser’ from Kerschbaumer et al. (2014).

**Table 1:** Information about sample size of investigated species and populations of *Tropheus*

Sampling site	Code	Habitat situation	Species	Color morph	Sample size (males/females)			
					Angular	Lacrymale	Preopercle	Quadrate
Ikola 1	IKA1	Non-sympatric	*T. moorii*	T.“Ikola”	28 (10/18)	17 (0/17)	30 (12/18)	29 (11/18)
Ikola 2	IKA2	Non-sympatric	*T. moorii*	T.“Ikola”	36 (18/18)	36 (19/17)	33 (16/17)	33 (16/17)
Ikola 3	IKA3	Non-sympatric	*T. moorii*	T.“Ikola”	48 (24/24)	48 (24/24)	46 (25/21)	49 (25/24)
Mahale	KFS1	Sympatric	*T. moorii*	T.“Kirschfleck”	31 (22/9)	30 (22/8)	31 (22/9)	31 (22/9)
	TPS1		*T. polli*		29 (14/15)	29 (13/16)	30 (14/16)	30 (14/16)
Mabilibili	KFS2	Sympatric	*T. moorii*	T.“Kirschfleck”	30 (16/14)	28 (16/12)	29 (15/14)	29 (16/13)
	TPS2		*T. polli*		30 (14/16)	30 (14/16)	30 (14/16)	30 (14/16)
South of Isonga	IKS3	Sympatric	*T. moorii*	T.“Ikola”	30 (16/14)	30 (16/14)	30 (16/14)	30 (16/14)
	TPS3		*T. polli*		29 (16/13)	32 (16/16)	31 (15/16)	30 (15/15)
North of Kekese	IKS4	Sympatric	*T. moorii*	T.“Ikola”	30 (11/19)	29 (11/18)	28 (12/16)	28 (10/18)
	TPS4		*T. polli*		29 (13/16)	30 (13/17)	30 (13/17)	30 (12/18)
Kekese	IKS5	Sympatric	*T. moorii*	T.“Ikola”	34 (17/17)	41 (22/19)	40 (21/19)	39 (21/18)
	TPS5		*T. polli*		38 (28/10)	38 (28/10)	38 (28/10)	38 (28/10)

### Data acquisition

Due to the much more work-intensive maceration process and microscopic photography, a subsample of Kerschbaumer et al. (2014) was considered here. After scanning anesthetized fish for studying overall morphology, about 450 fish were killed by an overdose of clove oil, stored in 70% EtOH after being preserved in 10% formalin and ascending concentrated alcohol solutions. Heads were cut off behind the fifth dorsal fin ray and the eyes and scales were removed. All specimens were cleared and stained with alcian blue and alizarin red using a modification of the procedure described by Potthoff (1984). The next step was disarticulation with Enzyrim^®^ (Grundmann & Roetzscher, 2000) following the standard recipe with an incubation overnight at 55°C. Digital images of the following viscerocranial elements were taken: Articular (Aa), lacrymale (Lac) preopercle (Pop) and quadrate (Qd).

**Fig. 2:**
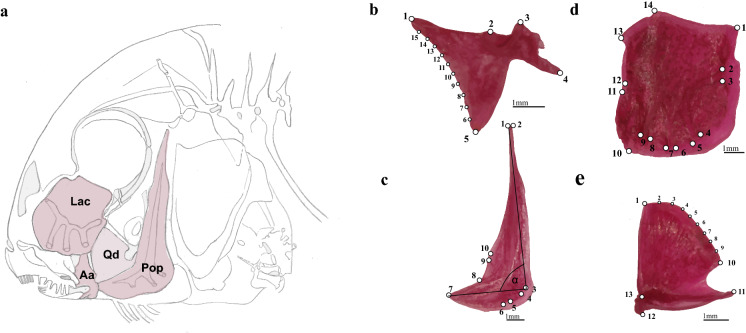
**a** Lateral view and location of investigated bones in the head of *Tropheus* (Lac lacrymale; Qd quadrate, Aa articular; Pop preopercle). Dissected bones (**b**) articular, (**c**) preopercle, (**d**) lacrymale, (**e**) quadrate with positions of landmarks, semilandmarks and other measurements. Detailed description of positions of landmarks and semilandmarks are given in Supplementary Table S1

The lacrymale is a plate-shaped bone between the eye and the upper jaw: it is the most rostral and by far the largest of the infraorbital bones, which border the ventral half of the eye (Barel & Kramers, 1977). Furthermore, we compared the shape of the articular. Its anterior process fits into the ventral process of the dentary. The quadrate articulates with and acts as the pivot for the angular bone of the lower jaw. A posterior, preopercular process features a groove where the preopercle articulates. The fourth bone we chose to study is the preopercle, a boomerang-shaped bone with an upper vertical margin, sometimes called the upper limb, and a lower horizontal edge, called the lower limb; the two limbs meet at the angle of the preopercle. All bones are quite flat, such that 2D landmarks are appropriate for their analyses. Figure 2 shows schematic drawing of *Tropheus’s* head with focus on investigated bones. Sampling details are given in Table 1.

All elements were taken from the left body side except for very few specimens where this was damaged. Digital images were taken in standardized position with an Olympus digital camera (Olympus E-1) mounted on an Olympus SZX-ILLB2-200 binocular eyepiece. Coordinates of landmarks and semilandmarks were digitized using TpsDig 2.05 (Rohlf, 2005). To detect shape variation in the curved part of articular and quadrate, we employed the program MakeFan 7.0 (Sheets, 2005). With this software, one can produce equidistant fans on relevant parts of objects to place evenly spread semilandmarks. Semilandmarks were slid to minimize the bending energy (Gunz & Mitteroecker, 2013) using TpsRelw Vers.1.76 (Rohlf, 2010). For the other two bones, there are no areas like curves, so we only digitized landmarks. For position and description of landmarks and semilandmarks, please check Table S1 and Fig. 2.

### Data analysis

We used a geometric morphometric approach based on Procrustes methods (Bookstein, 1996; Dryden & Mardia, 1998; Mitteroecker & Gunz, 2009). We carried out analyses on the residuals from a regression of shape on centroid size. Those residuals are shape values from which the effects of size have been removed. For more detailed information, read Klingenberg (2016). We analyzed shape differences through principal component analysis (PCA). PCA, where groups are not defined a priori, allows us to have an insight in the shape variation of bones among the different populations. We used PCA considering individuals to gather variation within populations and we did it on population mean shapes. In a following step, we distinguished males and females in PCA to look for sexual dimorphism in single bones. We computed population and sex-specific mean shapes and analyzed differences for each bone separately. Morphometric analyses were done in MorphoJ (Klingenberg, 2016).

To quantify statistical differences in shape of investigated viscerocranial bones between species, and between groups within species, we used the Procrustes ANOVA evaluated for significance with the *F* test (Goodall, 1991). This distance-based ANOVA uses Procrustes distances among specimens but is statistically equivalent to a regular analysis of variance. We also evaluated a nested model considering different interactions of species, habitat scenario, color morph and sex. Significance testing was obtained through a residual randomization permutation procedure involving 1000 permutations (Collyer et al., 2015).

To evaluate the amount of shape variation within species as well as within populations, we estimated morphological disparity by measuring Procrustes variance, which is the dispersion of all observations around a mean shape for the group (Zelditch et al., 2012). We also tested for statistical differences between groups running a randomized permutation procedure (1000 permutations). ANOVA and analyses of morphological disparity were performed in RStudio Version 1.3.1093 (RStudio Team, 2020) using the *geomorph* library 3.3.1 (Collyer & Adams, 2018, 2020; Adams et al., 2020).

For one bony element, namely preopercle we additionally measured the angle between its two limbs using CoordGen8 (Sheets, 2017) and compared measurements among males and females by doing a two-sample *t* test in R Version R-3.5.1 (R Core Team, 2018) using the library *stats,* which is part of R. To check for correlation of population shape differences with genetic divergence among populations, we generated regressions of Procrustes distances on FSTs (published in Kerschbaumer et al., 2014).

## Results

**Fig. 3:**
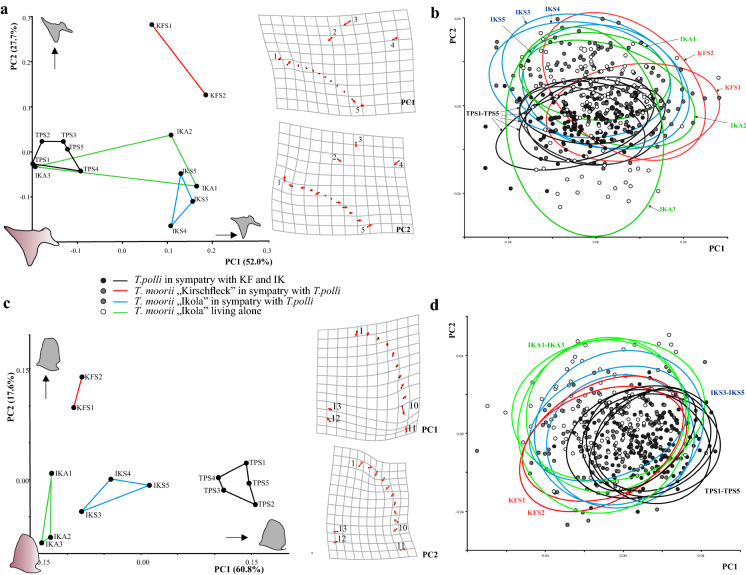
**a** Scatter plots of the 13 population mean shapes and **b** individual scores with 90% confidence interval ellipses of the first two principal components for articular and **c** and **d** for quadrate shape. (For abbreviations of populations see legend of Fig. 1)

**Fig. 4:**
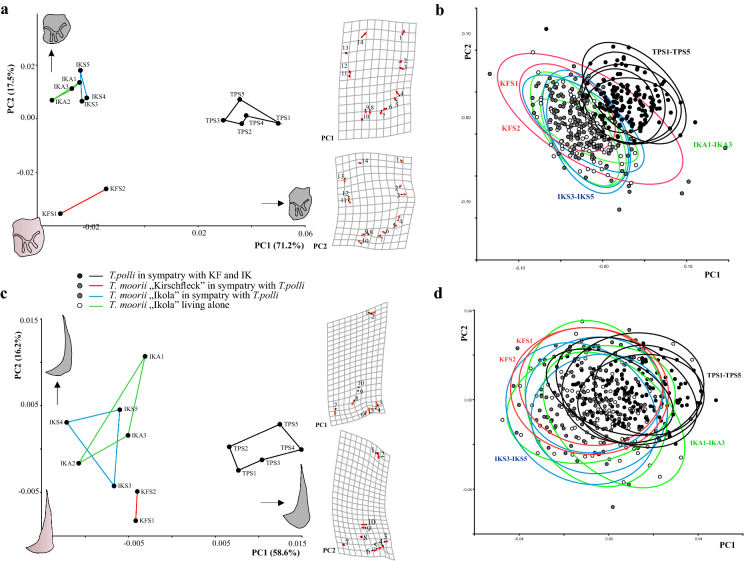
**a** Scatter plots of the 13 population mean shapes and **b** individual scores with 90% confidence interval ellipses of the first two principal components for preopercle and **c** and **d** for lacrymale shape. (For abbreviations of populations see legend of Fig. 1)

Standard length (SL) of fish specimens is not a distinguishing factor among populations of the two scenarios with and without a congeneric competitor. We observed different ranges of SL but the mean SL is quite consistent in all investigated populations (Fig. S1). In all four bones, the first two components of PCA describe more than 70% of variation in the dataset. Species discrimination is clear for quadrate (Fig. 3c), lacrymale (Fig. 4a) and preopercle (Fig. 4c) since *T. polli* populations (TPS1-TPS5) show clearly different mean shapes to *T. moorii* populations. All bones show separation among *T. moorii* and *T. polli* along the leading eigenvector, PC1. For articular, most pronounced shape changes along PC1 are concentrated at the articulation facet of the suspensorium (Fig. 3a), and for quadrate there is a lot of change in the curved part between LM 1 and 10 and at the caudal-most point of upper plane part of the quadrate (Fig. 3c). The difference of lacrymale’s shape among the two species seems to be a higher and more slender lacrymale in *T. polli* (Fig. 4a). For preopercle, we can see an enlarging of its lower part in *T. polli* populations (Fig. 4c). Within *T. moorii* populations, articular and preopercle show fewest shape differences (Figs. 3b and 4d).

Morphospace patterns suggest that only the quadrate differs among non-sympatric and sympatric *T. moorii* populations (Fig. 3c, d). IKA1, IKA2 and IKA3 have lower PC 1 scores and can be separated from the five sympatric populations, IKS3, IKS4, IKS5, KFS1 and KFS2 in their mean shape of this bone. Mean quadrate shapes of the *T. polli* populations are located at high PC1 scores and sympatric populations tend to go to the same direction (Fig. 3c). Scatterplot concerning individuals indicate this pattern (Fig. 3d). Shape differences according to PC1 are located at the pivot for articular and LM 10, but this position is hard to define in two dimensions in terms of connection to other bones and structures. Scatterplot indicates that the shape of the articular bone seems to be very variable in one non-sympatric population, namely IKA3.

**Fig. 5:**
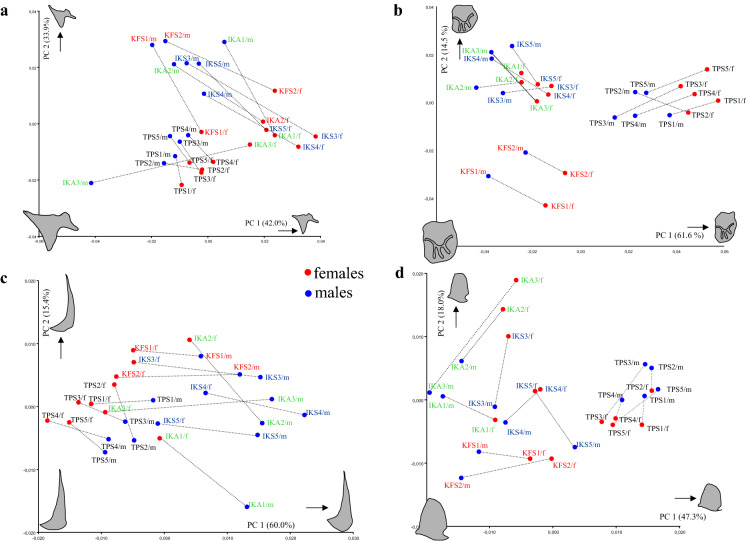
Scatter plots of population and sex-specific mean shapes (blue dots for males and red dots for females) for **a** articular, **b** lacrymale, **c** preopercle and **d** quadrate with shape changes according to PC axis 1 and 2. (For abbreviations of populations see legend of Fig. 1)

Sex-specific mean shapes in all four visceral bones differentiate males and females, whereupon quadrate shows no consistent pattern of differentiation (Fig. 5). The strongest sexual dimorphism is manifested in preopercle, orientated in one particular direction, namely PC1 (Fig. 5c). The horizontal arm of the preopercle in males is broader and its most rostrad point turns more upward, as demonstrated by the shape changes towards a warped outline drawing of the bony element at the maximum of axis PC1 axes. Angle measurement between the vertical and horizontal limb of the preopercle confirmed this finding in that males tend to show at least a one-degree smaller angle among those two limbs (two-sample *t* test; *P* < 0.0001).

Procrustes ANOVA reveals that factors, like species, sympatric/non-sympatric living, population and color morph, independent from one another significantly determine morphology of all four osteological units (Table 2). Only quadrate shows no significant difference in shape, when sex is considered as factor (*P* = 0.115). For the nested design, where interaction of different factors is evaluated, we have again significant *P* values for all four bones, except one insignificant value for the factor species/color morph/sex in quadrate (Table 3).

**Table 2:** Procrustes ANOVA evaluating shape of the four bony elements between species, sympatric/non-sympatric habitat situations, populations, color morphs, sex and results of Nested ANOVA assessing shape between living situations, sex and color morphs within species

	Df	SS	MS	*R*^2^	*F*	*Z*	Pr (> *F*)
Articular							
Species	1	0.03878	0.038781	0.04537	19.914	5.2487	0.001*
Sympatric/non-sympatric	1	0.00863	0.0086341	0.0101	4.2756	2.752	0.003*
Population	12	0.15625	0.013021	0.1828	7.6057	11.156	0.001*
Color morph	2	0.06575	0.032873	0.07692	17.415	7.0007	0.001*
Sex	1	0.07383	0.073827	0.08637	39.611	6.2962	0.001*
Total	420	0.85476					
Species:sympatric/non-sympatric	1	0.01998	0.019982	0.02338	10.493	4.2407	0.001*
Species:sympatric/non-sympatric:sex	3	0.10275	0.034249	0.1202	20.502	8.9196	0.001*
Species:colormorph:sex	3	0.09369	0.03123	0.10961	18.639	8.3268	0.001*
Lacrymale							
Species	1	0.37513	0.37513	0.14771	72.098	8.6307	0.001*
Sympatric/non-sympatric	1	0.1076	0.107596	0.04237	18.405	6.0908	0.001*
Population	12	0.58094	0.048412	0.22876	10.01	14.168	0.001*
Color morph	2	0.4555	0.227749	0.17936	45.351	10.307	0.001*
Sex	1	0.08026	0.080263	0.0316	13.577	5.5278	0.001*
Total	417	2.53959					
Species:sympatric/non-sympatric	1	0.01854	0.01854	0.0073	3.5854	2.9807	0.002*
Species:sympatric/non-sympatric:sex	3	0.1046	0.03487	0.04119	7.037	6.9982	0.001*
Species:colormorph:sex	3	0.11205	0.03735	0.04412	7.8031	7.2641	0.001*
Preopercle							
Species	1	0.03836	0.038364	0.10372	49.064	6.772	0.001*
Sympatric/non-sympatric	1	0.00961	0.0096114	0.02598	11.311	4.4971	0.001*
Population	12	0.06675	0.0055627	0.18046	7.5785	10.763	0.001*
Color morph	2	0.04387	0.0219342	0.1186	28.458	7.9595	0.001*
Sex	1	0.0162	0.0161979	0.04379	19.418	5.4055	0.001*
Total	425	0.3699					
Species:sympatric/non-sympatric	1	0.00439	0.00439	0.01187	5.6767	3.3192	0.001*
Species:sympatric/non-sympatric:sex	3	0.02047	0.006824	0.05534	9.3451	6.8227	0.001*
Species:colormorph:sex	3	0.0212	0.007067	0.05732	9.737	6.8185	0.001*
Quadrate							
Species	1	0.003*2	0.003*2019	0.00629	2.6954	1.9157	0.026*
Sympatric/non-sympatric	1	0.003*85	0.003*8533	0.00757	3.2479	2.2186	0.009*
Population	12	0.05027	0.0041891	0.09871	3.7877	7.311	0.001*
Color morph	2	0.0082	0.0041009	0.01611	3.4785	3.1254	0.001*
Sex	1	0.00204	0.002044	0.00401	1.7168	1.1545	0.115
Total	427	0.50925					
Species:sympatric/non-sympatric	1	0.00513	0.0051255	0.01006	4.3487	2.6143	0.002*
Species:sympatric/non-sympatric:sex	3	0.00673	0.0022424	0.01321	1.9148	1.9258	0.027*
Species:colormorph:sex	3	0.00504	0.001*6785	0.00989	1.428	1.1813	0.114

*P values significant at the 5% level

**Table 3:** Bone shape variance (scaled by 10^3^) within species, color morph and habitat scenario for each bony element. Pairwise comparisons of disparity are given in Table S4 in supplementary

	Articular	Lacrymale	Preopercle	Quadrate
*Tropheus* “Ikola” non-sympatric	2.460	5.695	0.977	1.227
*Tropheus* “Ikola” sympatric	2.279	6.296	0.862	1.316
*Tropheus* “Kirschfleck” sympatric	2.367	7.723	0.921	1.162
*T. polli* sympatric	1.439	5.577	0.778	1.096

**Fig. 6:**
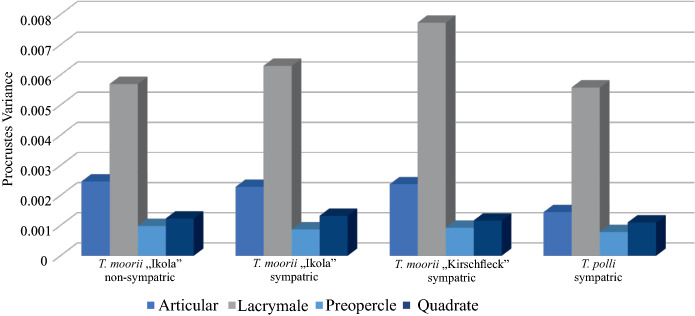
Graphical presentation of different bone shape disparities

Figure 6 displays that shape disparity is very variable among the four bony elements. Disparity (Procrustes variance) is particularly higher in lacrymale as in the other three bones. For articular, analysis reveals that Procrustes variance in all *T. polli* populations is significantly lower than in *T. moorii* populations (Fig. 6, Table S2). IKA3 shows the highest disparity in articular shape in all *Tropheus* populations (Table S2). Comparison of Procrustes distances and genetic distances among *Tropheus* “Ikola” populations revealed no correlation in all four bones (Fig. S2).

## Discussion

It has been argued that habitat partitioning could be the first step in adaptive radiations (Streelman & Danley, 2003), and LT with its rocky shorelines provides several opportunities for cichlids to diversify along a habitat gradient that is shaped by increasing water depth affecting the accessible resources (Seehausen, 2015). It was shown that morphology of the trophic apparatus, including the lower pharyngeal jaw, strongly correlates with the feeding mode. Thus, adaptation to the available niches is a prominent driver of diversification in cichlid fishes (Liem, 1980). While there are several studies dealing with the correlation of trophic morphology and dietary specialization (e.g., Liem, 1978; Yamaoka, 1983; Barluenga et al., 2006; Hellig et al., 2010; Takahashi & Koblmüller, 2011), there are almost no studies published on the adaptive evolution of viscerocranial bones at the population level.

Muschick et al. (2012) showed that parallel adaptation to the same niche produced convergent morphologies, even within a single adaptive radiation in LT so that its species assemblage contains sets of convergent forms that sometimes live in sympatry. A considerable number of seeding lineages radiated in parallel and in response to the same external drivers. Convergent evolution appears to be particularly frequent in this species-rich community, leading to similar species pairs that coexist or at least overlap in the same niche. These eco-morphological equivalents both comprise species of distantly related lineages and sets of closely related species assigned to the same clade. For both levels of divergence, ecologically equivalent species were suggested to have evolved in allopatry, and subsequent secondary contact triggered by (repeated) lake level fluctuations, termed species pump evolution (Rossiter & Kawanabe, 2000). In a very recent study (Irisarri et al., 2018), novel evidence for hybridization at the onset of the Tanganyika species flock was found. This event might have boosted adaptive radiation of Tanganyika cichlids and jaw development was found to bear a strong signal of introgression.

The endemic Lake Tanganyika tribe Tropheini diversified into a wide variety of trophic niches in littoral habitats, ranging from predators to algae scrapers. Two clades, the genera *Petrochromis* and *Tropheus*, diversified further allopatrically, after colonizing all available shallow littoral rock habitats throughout the lake. While data are so far scarce for *Petrochromis*, the phylogeographic sub-structuring has been thoroughly analyzed for the genus *Tropheus*, of which 6 nominal species and about 120 geographical variants (color morphs) have been described (Poll, 1986; Schupke, 2003; Konings, 2013). *Tropheus*, when allopatric, hold a stable and equivalent ecological niche position in the species community of rock and cobble shores with their complex and fine-scaled interactions. Even if particular environmental parameters vary among shores and adaptive microevolutionary changes seem probable, all *Tropheus* populations are ecologically stabilized within their fundamental niche via coevolutionary interactions with the other species present (Van Valen, 1973). In fact, the relatively small scope of morphological change observed suggests stabilizing selection which is expected in mature stages of adaptive radiation (Greenwood, 1984). This lake-wide allopatric setting is complemented by relatively few cases of sympatry of more than one *Tropheus* species, making it possible to directly address potential consequences of ecological character displacement enforced by an eco-morphologically equivalent competitor. In all cases of congeneric sympatry, one (or two) *Tropheus* is (are) forced to deeper sections of the habitat by the superior competitor. The study of Conith et al. (2020) concerning a Lake Malawi cichlid complex studied shape of four bones involved in feeding in populations that inhabit deep versus shallow habitats. They found no difference in disparity, rates in morphological evolution, or the pattern of modularity between members residing different depth and suggested that conserved patterns of modularity permit the evolution of divergent morphologies and may ease shifts between habitats.

We know that habitat choice along a depth gradient correlates with large differences in light, temperature, wave action, diet and oxygen that lead to subsequent adaptive changes in many aspects. These may be exposure to different predators or increasing lack of bird predators at greater water depth. It may also concern adaptations in the sensory system such as differences in eye diameter found in Kerschbaumer et al. (2014) or shifts in the spectral sensitivity patterns in rhodopsin (Sugawara et al., 2005; Nikaido et al., 2014) or in other aspects of the sensory system (Seehausen, 2015). Previous studies on the overall body morphology of *Tropheus* populations point to particular adaptive features, possibly triggered by water depth, light, bird predation and intensity of wave action (Kerschbaumer et al., 2014).

In this study, we chose particular bones related to the shape of the viscerocranium, to find out if, and to which extent, bones would be ecologically informative concerning the depth-shift. Interestingly, only the quadrate shows shape differences, among sympatric and non-sympatric populations in PCA. The remaining three bones are somewhat distinct but there is much more variation among populations and individuals, resulting in a great deal of overlap, so that neutral drift seems more likely. Wanek & Sturmbauer (2015) investigated morphological variation among species of the tribe Tropheini, which occupy several trophic niches, but mostly rocky habitats. They found that morphology mostly correlates with ecological parameters and in most cases, it is not reflecting phylogenetic relatedness. In their study, differences in shape can be attributed to three main characteristics, namely mouth position, mouth size and body depth. Indeed, the quadrate’s connection to bones, near mouth and eye, can be the reason for the presence of an ecological signal in this bony element. One disadvantage of investigating two-dimensional pictures of bones is that it is hardly possible to describe, precisely concrete shape variation at bones. We could only roughly locate differences on the bony elements. After ANOVA, we become aware that there are many more factors, which significantly influence shape of viscerocranial bones. Those factors are species, population, color morph and sex and it is quite difficult to isolate and discuss only one of them.

Next to morphological differentiation, morphological disparity plays an important role in our dataset. The lacrymale is by far the most variable bone in this study. The fact that the lacrymale bears such a big reservoir of shapes could relate to findings of Cooper et al. (2010) that the preorbital region represents an evolutionary module which can respond quickly to natural selection when fish colonize new lakes. Furthermore, number and size of sensory pores on these bones were associated with noise sensitivity (Bleckmann & Zelick, 1993). There also may be a functional connection between lacrymale shape, auditory sensitivity and even acoustic communication. Further studies are needed to address this question.

Concerning morphological disparity in bones between *Tropheus* species, we see the same trend as for whole body shape in *Tropheus* (Kerschbaumer et al., 2014), namely that *T. polli* populations are less variable in their bone shapes than *T. moorii* populations, and that may also be a result of stabilizing selection. Another interesting finding was that the non-sympatrically living population IKA3 shows very diverse shapes of their articular bone (Fig. 3b, Table S2). This substantial morphological variety in one viscerocranial bone within one population might be related to the geographical position of IKA3 at the outermost border of our sampling area (Fig. 1) but this might be addressed in another study.

Unexpectedly, we revealed strong signals of sexual dimorphism in three out of the four studied bones. Articular, preopercle and lacrymale show strong distinction and congruent patterns of discrimination among populations. We know that size and shape of the cichlid head differ between sexes in some maternally mouthbrooding species and sexual dimorphism appears to be related to a larger buccal cavity in females (Takahashi & Hori, 2006; Herler et al., 2010). Thus, it is not astonishing that our investigated bones, which border the buccal cavity (Fig. 2), show sex-specific differences.

We know that cichlids share similar structures and mechanical functions in their lower jaw. There are many studies, dealing with mechanical properties based on simple levers being a benefit in exploiting different types of prey (Barel, 1983; Wainwright & Richard, 1995; Westneat, 1995; Albertson et al., 2003a). It was shown that variation in these lever-like elements always induce consequences which are putatively adaptive (Albertson et al., 2005). Even though our results are based on individual bony elements and we cannot discuss lever consequences for the mechanics of mouth opening and feeding behavior, we want to emphasize the fact that we compare structures within one species and niche. It is exactly these subtle differences that may represent the first very small steps towards morphological adaptation to a different microhabitat. Our comparison of Procrustes distances and genetic distances among *Tropheus* “Ikola” populations and the absence of their correlation go beyond previous reports (Kerschbaumer et al., 2014), showing that there is a strong indication of the action of differential selective processes in *Tropheus* populations.

In summary, analyses of the viscerocranial bones: articular, lacrymale, preopercle and quadrate by means of geometric morphometrics, give us a lot of information about the potential of every bone in terms of (micro-)evolution. The presence of subtle, but consistent morphological differences in single bony elements, among several sympatric and non-sympatric *Tropheus* populations, adds to the growing body of evidence that the preorbital region played a key role in the impressive radiation of LT cichlid fishes. Those viscerocranial adaptations allow an optimal exploitation of ecological niches and those phenotypic trajectories may be central to the extensive adaptive radiation of East African cichlids.

## Supplementary Information

Below is the link to the electronic supplementary material.

Supplementary material 1 (DOCX 52 kb)
