# Supplementary material for: Microevolutionary change in viscerocranial bones under congeneric sympatry in the Lake Tanganyikan cichlid genus Tropheus
Source: Hydrobiologia. 2021 Feb 22;848(16):3639–53. doi: 10.1007/s10750-021-04536-7 (PMC8550039; doi:10.1007/s10750-021-04536-7)
Supplement: Supplementary file 1 — Supplementary material 1 (DOCX 52 kb) [file 10750_2021_4536_MOESM1_ESM.docx]

**Table S1.** Definitions of landmarks and semilandmarks

| **Element** | **Landmarks** | **Descriptions** |
| --- | --- | --- |
| Articular (lateral) | 1 | Dorsal tip of the articular process |
|  | 2 | Dorsal process of the suspensoriad articulation facet |
|  | 3 | Postarticulation process (of suspensoriad articulation facet) |
|  | 4 | Retroarticular process |
|  | 5 | Tip of the rostral articular process |
|  | 6 to 15 | Semilandmarks |
| Preopercle (lateral) | 1 | Anterior-most and dorsal-most tip of the preopercle |
|  | 2 | Posterior-most and dorsal-most tip of the preopercle |
|  | 3 to 6 | Openings to the lateral-line system |
|  | 7 | Rostral-most point of the lower part of preopercle |
|  | 8 | Rostral most vertex between the vertical and horizontal limb of the preopercle |
|  | 9.10 | Openings to the lateral-line system |
| Lacrymal | 1 | Posterior-most and dorsal-most tip of the lacrymal |
|  | 2 to 9 | Openings to the lateral-line system |
|  | 10 | Rostroventral-most point of the lacrymal |
|  | 11.12 | Openings to the lateral-line system |
|  | 13 | Rostrodorsal-most point of the lacrymal |
|  | 14 | Dorsal-most point of th lacrymal |
| Quadrate | 1 | Outside-most dorsal point of the quadrate |
|  | 2 to 9 | Semilandmarks |
|  | 10 | Caudal-most point of upper plane part of the quadrate |
|  | 11 | Caudal-most tip of lower arm of the quadrate |
|  | 12 | Rostroventral-most point of the articulation process |
|  | 13 | Groove between the rostral upper plane part and the articulation process of the quadrate |

**Table S2.** Pairwise comparisons of Procrustes variance between populations. Upper triangle values are *P*-values associated with pairwise differences (1,000 permutations) concerning the bone **articular**. Values in the lower triangle are *P*-values derived from the analysis of **lacrymale**. P-values in bold are significant at the 5% level.

|  | IKA1.A | IKA2.A | IKA3.A | IKS3.S | IKS4.S | IKS5.S | KFS1.S | KFS2.S | TPS1.S | TPS2.S | TPS3.S | TPS4.S | TPS5.S |
| --- | --- | --- | --- | --- | --- | --- | --- | --- | --- | --- | --- | --- | --- |
| IKA1.A | 1 | 0.36 | **0.036** | 0.254 | 0.85 | 0.456 | 0.771 | 0.754 | 0.242 | **0.027** | **0.006** | **0.001** | **0.017** |
| IKA2.A | 0.124 | 1 | **0.001** | **0.037** | 0.456 | 0.865 | 0.211 | 0.209 | 0.754 | 0.156 | **0.035** | **0.007** | 0.101 |
| IKA3.A | 0.284 | 0.322 | 1 | 0.391 | **0.01** | **0.002** | 0.058 | 0.053 | **0.001** | **0.001** | **0.001** | **0.001** | **0.001** |
| IKS3.S | 0.063 | 0.355 | 0.098 | 1 | 0.159 | **0.04** | 0.36 | 0.371 | **0.016** | **0.001** | **0.001** | **0.001** | **0.001** |
| IKS4.S | 0.534 | 0.204 | 0.554 | 0.089 | 1 | 0.568 | 0.61 | 0.604 | 0.303 | 0.05 | **0.005** | **0.003** | **0.024** |
| IKS5.S | 0.189 | 0.54 | 0.641 | 0.17 | 0.358 | 1 | 0.293 | 0.287 | 0.626 | 0.115 | **0.025** | **0.005** | 0.088 |
| KFS1.S | 0.13 | 0.838 | 0.382 | 0.258 | 0.217 | 0.679 | 1 | 0.985 | 0.128 | **0.01** | **0.002** | **0.002** | **0.008** |
| KFS2.S | 0.053 | 0.205 | 0.072 | 0.487 | 0.055 | 0.112 | 0.169 | 1 | 0.132 | **0.01** | **0.002** | **0.001** | **0.01** |
| TPS1.S | 0.078 | 0.613 | 0.151 | 0.629 | 0.122 | 0.291 | 0.468 | 0.305 | 1 | 0.287 | 0.075 | **0.029** | 0.242 |
| TPS2.S | 0.386 | 0.252 | 0.792 | 0.112 | 0.762 | 0.496 | 0.294 | 0.077 | 0.149 | 1 | 0.453 | 0.192 | 0.922 |
| TPS3.S | 0.752 | 0.178 | 0.368 | 0.065 | 0.711 | 0.247 | 0.156 | **0.034** | 0.094 | 0.521 | 1 | 0.631 | 0.491 |
| TPS4.S | 0.253 | 0.388 | 0.892 | 0.134 | 0.489 | 0.77 | 0.447 | 0.094 | 0.19 | 0.701 | 0.324 | 1 | 0.249 |
| TPS5.S | 0.348 | 0.294 | 0.917 | 0.116 | 0.636 | 0.581 | 0.35 | 0.078 | 0.155 | 0.894 | 0.438 | 0.805 | 1 |

**Table S3.** Pairwise comparisons of Procrustes variance between populations. Upper triangle values are *P*-values associated with pairwise differences (1,000 permutations) concerning the bone **preopercle**. Values in the lower triangle are *P*-values derived from the analysis of **quadrate**. P-values in bold are significant at the 5% level.

|  | IKA1.A | IKA2.A | IKA3.A | IKS3.S | IKS4.S | IKS5.S | KFS1.S | KFS2.S | TPS1.S | TPS2.S | TPS3.S | TPS4.S | TPS5.S |
| --- | --- | --- | --- | --- | --- | --- | --- | --- | --- | --- | --- | --- | --- |
| IKA1.A | 1 | 0.886 | 0.835 | 0.112 | 0.601 | 0.177 | 0.355 | 0.924 | **0.006** | 0.079 | 0.183 | 0.335 | 0.442 |
| IKA2.A | 0.289 | 1 | 0.707 | 0.089 | 0.698 | 0.093 | 0.296 | 0.957 | **0.006** | 0.061 | 0.12 | 0.274 | 0.375 |
| IKA3.A | 0.552 | 0.07 | 1 | 0.145 | 0.435 | 0.189 | 0.376 | 0.745 | **0.004** | 0.082 | 0.203 | 0.392 | 0.526 |
| IKS3.S | 0.238 | **0.034** | 0.455 | 1 | **0.048** | 0.827 | 0.564 | 0.101 | 0.222 | 0.779 | 0.843 | 0.55 | 0.383 |
| IKS4.S | 0.426 | 0.076 | 0.74 | 0.723 | 1 | 0.058 | 0.152 | 0.681 | **0.002** | **0.027** | 0.064 | 0.147 | 0.192 |
| IKS5.S | 0.553 | 0.629 | 0.206 | **0.049** | 0.158 | 1 | 0.673 | 0.132 | 0.127 | 0.607 | 0.988 | 0.688 | 0.489 |
| KFS1.S | 0.817 | 0.393 | 0.433 | 0.158 | 0.323 | 0.716 | 1 | 0.284 | 0.069 | 0.374 | 0.712 | 1 | 0.818 |
| KFS2.S | 0.495 | 0.704 | 0.198 | 0.067 | 0.16 | 0.946 | 0.671 | 1 | **0.005** | 0.065 | 0.154 | 0.314 | 0.388 |
| TPS1.S | 0.421 | 0.84 | 0.131 | **0.047** | 0.123 | 0.8 | 0.533 | 0.848 | 1 | 0.345 | 0.138 | 0.072 | **0.038** |
| TPS2.S | 0.158 | 0.72 | **0.041** | **0.006** | **0.039** | 0.396 | 0.224 | 0.467 | 0.594 | 1 | 0.599 | 0.373 | 0.239 |
| TPS3.S | 0.425 | 0.824 | 0.15 | 0.051 | 0.125 | 0.821 | 0.571 | 0.902 | 0.96 | 0.55 | 1 | 0.699 | 0.517 |
| TPS4.S | 0.274 | 0.971 | 0.066 | **0.024** | 0.066 | 0.593 | 0.394 | 0.676 | 0.807 | 0.749 | 0.784 | 1 | 0.82 |
| TPS5.S | 0.937 | 0.232 | 0.587 | 0.236 | 0.456 | 0.464 | 0.81 | 0.453 | 0.339 | 0.127 | 0.346 | 0.248 | 1 |

**Table S4.** Pairwise comparisons of Procrustes variance (1,000 permutations) in (a) articular, (b) lacrymale, (c) preopercle and (d) quadrate between species and colormorphs. P-values in bold are significant at the 5% level.

| **(a)** | *Articular* | Ikola.A | Ikola.S | Kirschfleck.S | Polli.S |
| --- | --- | --- | --- | --- | --- |
|  | Ikola.A | 1 | 0.332 | 0.638 | **0.001** |
|  | Ikola.S |  | 1 | 0.693 | **0.001** |
|  | Kirschfleck.S |  |  | 1 | **0.001** |
|  | Polli.S |  |  |  | 1 |
|  |  |  |  |  |  |
|  |  |  |  |  |  |
| **(b)** | *Lacrymale* | Ikola.A | Ikola.S | Kirschfleck.S | Polli.S |
|  | Ikola.A | 1 | 0.542 | 0.087 | 0.893 |
|  | Ikola.S |  | 1 | 0.211 | 0.424 |
|  | Kirschfleck.S |  |  | 1 | 0.057 |
|  | Polli.S |  |  |  | 1 |
|  |  |  |  |  |  |
| **(c)** | *Preopercle* | Ikola.A | Ikola.S | Kirschfleck.S | Polli.S |
|  | Ikola.A | 1 | 0.126 | 0.538 | **0.002** |
|  | Ikola.S |  | 1 | 0.524 | 0.231 |
|  | Kirschfleck.S |  |  | 1 | 0.084 |
|  | Polli.S |  |  |  | 1 |
|  |  |  |  |  |  |
| **(d)** | *Quadrate* | Ikola.A | Ikola.S | Kirschfleck.S | Polli.S |
|  | Ikola.A | 1 | 0.421 | 0.565 | 0.17 |
|  | Ikola.S |  | 1 | 0.232 | **0.029** |
|  | Kirschfleck.S |  |  | 1 | 0.599 |
|  | Polli.S |  |  |  | 1 |


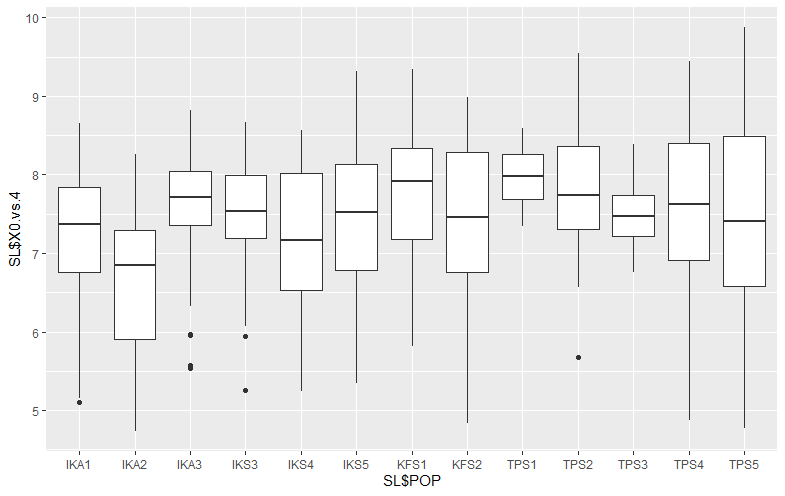


**Figure S1.** Boxplots showing standard length of specimens belonging to different populations. Measurements derived from the study of Kerschbaumer et al. (2014).

**Figure S2.** Regression of pairwise F_st_ and Procrustes distance values. F_st_ on horizontal and Procrustes distance on vertical axis for different bones.
